# Mycotoxins in blood and urine of Swedish adolescents—possible associations to food intake and other background characteristics

**DOI:** 10.1007/s12550-019-00381-9

**Published:** 2019-12-14

**Authors:** Eva Warensjö Lemming, Andrea Montano Montes, Jessica Schmidt, Benedikt Cramer, Hans-Ulrich Humpf, Lotta Moraeus, Monica Olsen

**Affiliations:** 1 Risk Benefit Assessment Department, Swedish Food Agency, PO Box 622, 75126 Uppsala, Sweden; 2 Karolinska institute, Institute of Environmental Medicine, Box 210, 171 77 Stockholm, Sweden; 3 Westfälische Wilhelms-Universität Münster, Institute of Food Chemistry, Corrensstr. 45, 48149 Münster, Germany

**Keywords:** Deoxynivalenol, Ochratoxin A, 2’R-ochratoxin A, Enniatin B, Dietary recall

## Abstract

The exposure to mycotoxins of Swedish adolescents is currently unknown. The aim of the present study was to investigate the exposure to mycotoxins and their association with food intake, and background characteristics in adolescents of a national dietary survey. About 3000 school students (1000 from the 5th, 8th and 11th school years) were recruited for the survey. The participants completed Web-based questionnaires on food propensity, sociodemography and health, and a Web-based dietary recall. Spot urine and blood samples were collected from 1105 of the participants for mycotoxin biomarker analysis. Mycotoxins were analysed with multibiomarker methods in urine (HPLC-MS/MS) and serum (HPLC-MS/MS). Of the 35 different analytes in urine, the frequency of positive samples were the following: deoxynivalenol (DON, 4.8%), DON-15-β-D-O-glucuronide (DON-15GlcA, 9.1%), dihydro-citrinone (DH-CIT, 0.5%), HT-2-glucuronide (HT-2-3-GlcA, 0.1%) and ochratoxin A (OTA, 0.1%). Of the 27 different analytes in serum, OTA was detected in all samples, while 2’R-ochratoxin A (2’R-OTA) was found in 8.3% and enniatin B (EnB) in 99.2% of the samples. Exposure assessment calculations were performed on OTA from the serum concentration and on DON equivalents (DON eqv) from the urine concentration. All probable daily intake (PDI) estimates were below tolerable daily intakes, except for 1.6% of the participants for DON. The maximum PDI was 4.3 μg DON eqv/kg body weight and day. Consumption of cereal grain commodities was associated with levels of DON, EnB or OTA in biofluids. Serum OTA was also associated with intakes of raisins and coffee. Furthermore, coffee consumption correlated well with 2’R-OTA concentration in serum. In conclusion, exposure to mycotoxins in Swedish adolescents is common, but fortunately, high exposure was rare.

**Electronic supplementary material:**

The online version of this article (10.1007/s12550-019-00381-9) contains supplementary material, which is available to authorized users.

## Introduction

Humans are exposed to multiple mycotoxins via food consumption and from the environment, usually by occupational exposure. The most important route of exposure of the general population is intake of contaminated foods and the most frequently detected mycotoxins are deoxynivalenol (DON) and ochratoxin A (OTA) (Ali et al. 2016; Heyndrickx et al. 2015; Märtlbauer et al. 2009; Solfrizzo et al. 2014; Wallin et al. 2015; Viegas et al. 2019). In addition to OTA, the isomer 2’R-ochratoxin A (2’R-OTA), which is formed during thermal processing of coffee, is of relevance since it was previously found in all blood samples of coffee drinkers in average half the concentration of OTA (Cramer et al. 2015). Besides contaminated food samples, exposure can also occur by inhalation of bioaerosols and organic dust or by dermal contact (Degen 2011). In risk assessment of mycotoxins, food consumption data and occurrence data from the corresponding foods are normally used to estimate population exposure. However, such method cannot estimate the individual intake and hence biomarker-based methods are more and more used to assess dietary exposure from blood or urine concentrations. This includes the detection of the parent compounds (mycotoxins) and/or their main phase I and phase II metabolites (e.g. glucuronide or sulphate conjugates). Another advantage is that biomarker-based methods include all sources of exposure. Human biomonitoring in combination with dietary surveys can be a useful tool to confirm exposure of mycotoxins, to correlate exposure to certain food intake and to perform trend analyses. In addition, it can be an important tool to reveal influence of other factors such as differences in exposure due to socioeconomic or regional factors (Ali et al. 2016; Breitholtz et al. 1991; Chen et al. 2017; Mitropoulou et al. 2018; Pacin et al. 2008). However, despite those benefits, human biomonitoring is more useful in human health and dietary studies, than its use in exact exposure assessment of daily intake. Until now, exposure assessment of daily intake from blood or urine concentration remains difficult, unless the human toxicokinetics and inter-individual differences are better understood (Ali et al. 2016; Dietrich et al. 2005; Duarte et al. 2011).

Of the mycotoxins of interest, DON is rapidly absorbed, distributed, metabolized and excreted. DON-15-glucuronide (DON-15-GlcA) is the most prominent proposed metabolite of DON, followed by DON-3-glucuronide (DON-3-GlcA) with a constant ratio around 4/1 DON-15-GlcA/DON-3-GlcA (Vidal et al. 2018). However, we must mention that the structure of DON-15-GlcA has not been fully elucidated as no NMR data have been published, yet. The mean excretion rate of total DON, as free DON and phase I and II metabolites, was recently compiled by (Faeste et al. 2018) and a mean excretion ratio of 70% was derived. De-epoxy-DON (DOM-1) has also been detected in human urine (Heyndrickx et al. 2015; Mitropoulou et al. 2018) but not consistently (Papageorgiou et al. 2018). DOM-1 is a detoxification product formed by the gut microbiota (Gratz et al. 2013) which also can epimerize DON to 3-epi-DON.

In contrast to DON, the toxicokinetics of OTA is complex. There are big differences between species, and human studies reveal that there are high inter- and intra-individual variations in the metabolism and excretion of OTA (O'Brien et al. 2001; Studer-Rohr et al. 2000). Following a human study, a two-compartment toxicokinetic profile was proposed. In the model, a fast distribution and elimination phase is followed by a second slower elimination phase, resulting in an elimination half-life of 35 days (Studer-Rohr et al. 2000).

The Swedish Food Agency has previously published an extended evaluation of urinary multi-biomarker analysis of mycotoxins and metabolites among adults (*n* = 250) and in school children (*n* = 50) in the 5th school year (Mitropoulou et al. 2018). DON and OTA were the most commonly occurring mycotoxins in urine of both adults and children. Besides OTA and DON, zearalenone (ZEN) and its metabolites α- and β-zearalenol (α- and β-ZEL) were frequently detected in the urine samples. However, all participants were well below the group tolerable daily intake (TDI) for ZEN and its modified forms established by the European Food Safety Authority (EFSA 2016). Estimates of DON exposure in adults showed that 1.3% of the participants were above the TDI.

The primary aim of this paper was to investigate the exposure to mycotoxins among adolescents in Sweden. Further, the study investigated the association between serum and urinary concentrations of mycotoxins and food intake, and socioeconomic and geographical background characteristics.

## Materials and methods

### Study design and population

Riksmaten Adolescents 2016–2017 is a nationally representative cross-sectional dietary survey conducted by the Swedish Food Agency (SFA) in Sweden. The survey was conducted during the school year 2016/2017 between September and May (Moraeus et al. 2018). The recruitment of participants was done class-wise in the 5th, 8th and 11th school years. The survey methods included a Web-based dietary assessment, Web questionnaires to collect information on background and lifestyle factors, measured weight and height and assessment of physical activity. Blood and urine samples were collected in a sub sample and the majority of the survey data were collected from the study participants at the day of the school visit.

Altogether, 5145 pupils at randomly selected schools were invited to participate. In total, 3477 individuals (68%) participated in some stage of the survey and 3099 participants (60%) provided complete information on diet. In the subsample, the response rate was 55% overall and 1105 (46%) participants had provided blood and urine samples and complete diet information, with small differences between the school years.

### Blood and urine samples

The blood and urine samples were collected in non-fasting subjects in collaboration with the regional divisions for Occupational and Environmental Medicine in Sweden. The blood samples were immediately centrifuged and placed in a freezer together with the urine sample. All samples were subsequently stored at − 80 °C until transportation for biomarker analysis. Transportation was made by courier to the laboratory in Münster, Germany, and packed with dry ice (approx. 15 kg). The samples reached the laboratory frozen and within 24 h. At the laboratory, the samples were stored at − 18 °C until analysis.

### Ethical permits

Ethical approval for the survey was obtained from the Regional Ethical Review Board in Uppsala (No. 2015/190). All participants that provided blood and urine samples left written informed consent prior to participation. For children younger than 16 years, the legal guardian/s provided informed consent for participation as well.

### Mycotoxin analyses in spot urine and serum samples

For the analysis of mycotoxin-biomarkers in urine samples a validated rapid “dilute and shoot” (DaS)-HPLC-MS/MS approach was applied according to Gerding et al. (2015). The method was extended by including 12 additional analytes, so that 35 compounds of interest were analysed in a chromatographic run time of 13 min. Limits of detection in urine were determined in a range of 0.01 to 15 ng/mL (Electronic Supplement Material 1). The total DON equivalents (DON eqv), the sum of free DON and DON-15GlcA converted to DONeqv, were calculated by converting DON-15GlcA to DON eqv by its molar mass as described by (Warth et al. 2012). Additionally, 10 urine samples obtained in the previous biomonitoring study by Wallin et al. (2013) were analysed in this study in order to compare the results of both method results for the estimation of DON exposure. The method used in the Wallin et al. study was a single DON method, including β-glucuronidase digestion to calculate the sum of total DON as free and conjugated. That method also included an immunoaffinity cleanup. For comparison of results from the two studies, the total DON eqv in this study was compared by the total DON of the Wallin study.

Human serum samples were analysed by use of a validated rapid multi-mycotoxin HPLC-MS/MS approach for biomonitoring of 27 mycotoxins and metabolites according to Osteresch et al. (Osteresch et al. 2017). Chromatographic separation of all compounds was achieved in a total run time of 11.5 min, which enabled high-throughput analysis as well as a chromatographic separation of OTA and 2’R-OTA. Further information on chemicals, reagents, sample preparations and analysis can be found in the Electronic Supplement Material 2.

### Dietary assessment

Participants recorded their consumption of foods and beverages in a web-based method called RiksmatenFlexDiet (Moraeus et al. 2018). The consumption was recorded during 3 days, whereof days 1 and 3 were nonconsecutive and retrospective. The second day was the day of the school visit and the day when participants provided blood and urine samples and consecutive to day 1. Day 3 was a random day occurring 3–9 days after day 2. Participants with complete diet information have valid diet data from at least days 1 and 3, but most participants have valid information from all 3 days (96%). RiksmatenFlexDiet contains all information needed for the registration including a picture portion guide and a search tool to search for the foods and beverages consumed. The food list contained 778 typical foods and beverages and is connected to the Swedish national food composition database (version Riksmaten Adolescents 2016–2017) for the calculation of energy intake and intakes of whole grains and dietary fibre (g/day).

### Food consumption data

#### Cereals, cereal grains and DON, EnB and OTA

The cereals and cereal grains data (food groups, foods and raw agricultural components) that was used in the analysis have previously been associated with DON exposure. These were breads and cereal products, breads, oats, barley (whole grain), barley (sifted), corn, rice (whole grain), rice, rye, wheat (whole grain) and wheat (sifted). As DON is rapidly excreted from the body, the food consumption data from the day before (day 1) the blood and urine samples were drawn were used in the analyses. Consumption data from day 1 can reflect a direct effect on the biological samples. In addition, the intake of whole grains and dietary fibre on day 1 and reflecting usual (long-term) intake was included in the analyses. The same cereal variables were investigated in association with EnB and OTA.

#### Other food items or food groups and OTA or 2’R-OTA

OTA is associated with other food sources than cereals, and the following foods were also included in the analyses with OTA: coffee, tea, cocoa, müsli, nuts/seeds, fruit/nuts, grape juice, liver pâté, blood pudding, dried fruits, processed meats, salami, cheese (soft and hard), pulses (beans, peas), nuts (peanuts, pistachio), pork, and pork offal. Due to the long half-time elimination of OTA and 2’R OTA, consumption of contaminated food items over a month prior to blood sampling could be relevant. To model an approximation of a typical long-term intake the mean food consumption over the three recording days for each food item and individual was calculated.

### Other variables

Parental education was collected from the web-based questionnaire, which was included in RiksmatenFlex. Highest degree of either parent was used and five levels of education were classified into ≤ 12 years and > 12 years of education, hereafter referred to as household education.

### Censoring, adjustment methods

Left-censored observations, which are values below the limit of detection (LOD) and quantitation (LOQ) of the analytical method, were imputed with the substitution method, as suggested by the European Food Safety Authority (EFSA), for chemicals likely to occur in food (EFSA 2010). Following this guidance, three scenarios were estimated, the lower bound (LB), middle bound (MB) and upper bound (UB). Specifically, results below LOD (or LOQ) were given the value zero in LB. To calculate mean concentration in positive samples (Tables 1 and 3), the values between LOD and LOQ were assigned a fixed value of LOQ/2. LOD and LOQ values for the corresponding mycotoxins are mentioned under Tables 1 and 3. To correct for inter-individual variations in urine volume, mycotoxin levels were adjusted for urine density and creatinine prior to analysis. For density adjustment, the formula provided by Smith et al. (2012) was used.

**Table 1:** Background characteristics of the participants in the Riksmaten adolescents 2016–2017 study, divided by school year

School year	5	8	11
*N*	331	411	354
Proportion of girls (%)	50	56	62
Weight (kg)	44 ± 10	59 ± 11	68 ± 13
Age (years)	11.6 ± 0.4	14.5 ± 0.4	17.8 ± 0.7
Energy intake (kJ)	8200 ± 1700	9600 ± 2600	9330 ± 2400
Breads and cereals day 1 (g)	136 (65; 237)	199 (114; 298)	175 (76; 309)
Breads day 1 (g)	48 (6; 95)	65 (0; 114)	54 (0; 114)
Whole grain, day 1 (g)	16 (3; 43)	24 (6; 53)	19 (5; 53)
Fibre, day 1 (g)	16 (12; 22)	19 (13; 26)	18 (12; 25)
Raisins^1^(g)	0.20 ± 1.2	0.80 ± 5.0	0.30 ± 2.0
Coffee^1^ (g)	1.0 ± 14	10 ± 50	55 ± 148

Values are given in mean ± standard deviation and median and 25th and 75th percentiles

^1^Long-term intake, mean of the consumption on days 1, 2 and 3

*C*(corr) = [*C*(obs) × (1.022–1)]/[(*ρ* − 1)]

where *C*(corr) (ng/ml) is the adjusted concentration, *C*(obs) (ng/ml) is the observed unadjusted concentration, 1.022 is the average density in our dataset (*n* = 1096), and *ρ* is the specific density in each urine sample.

Urine density was preferred compared to creatinine due to the concerns regarding creatinine’s dependence on other factors such as body size and diet (Suwazono et al. 2005), but both methods were used for comparative reasons. The daily urinary creatinine clearance as a function of body mass, age and sex, using a Web-based calculator found at http://www.clinicalculator.com/english/nephrology/excrea/excrea.htm, was used to estimate the daily urinary excretion expressed as ng DON equivalents/mg creatinine. Samples with creatinine levels < 0.3 or > 3.0 mg/ml are deleted according to Cocker et al. (2011).

### DON exposure assessment

Exposure assessment calculations were performed on DON eqv (DON + DON-15GlcA), by converting DON-15-β-D-O-glucuronide (DON-15GlcA) by its molar mass. The urinary mycotoxin concentrations were used to calculate a provisional daily intake (PDI (ng/kg bw)) per individual using the formula derived from the formula reported by Solfrizzo et al. (2014):

$$ \mathrm{PDI}=\left[C\left(\mathrm{corr}\right)\times V/\left(\mathrm{bw}\times \mathrm{excretion}\kern0.34em \mathrm{rate}\right)\right]\times 100 $$

where *C*(corr) (ng/ml) is the mycotoxin concentration adjusted for urine density or creatinine, bw (kg) is the body weight, *V* (ml) is the urine volume, excretion rate (%)

Density was determined using a hand refractometer (Atago CO LTD), and creatinine was determined according to Mazzachi et al. (2000). For density-adjusted values, a mean daily urine volume of 1500 ml used for adults were used for students in the 8th and 11th school year and 1000 ml for children in the 5th school year. The urinary excretion rate of DON in humans used for the calculation was 72.3% (Turner et al. 2010), a value close to that reported in other studies (Ali et al. 2016; Faeste et al. 2018; Heyndrickx et al. 2015; Papageorgiou et al. 2018; Warth et al. 2012), and for comparative reasons, the same that we have used in previous studies (Mitropoulou et al. 2018; Wallin et al. 2013). For citrinin, an excretion rate of 40.2% was used (Ali et al. 2018).

### OTA exposure assessment

Exposure assessment of OTA was done by calculating PDI using two models suggested in the literature. Model 1 is the most commonly used for intake estimation from serum found in previous literature (Ali et al. 2018; Coronel et al. 2010; Duarte et al. 2011; Soto et al. 2016) and is built on a human study on the kinetics of OTA which was presented in a dissertation by Studer-Rohr (1995a). In the dissertation, Studer-Rohr compares renal and plasma clearance and concludes that other routes of excretion other than the renal route are negligible. The model was derived from the Klaassen equation which describes the relationship between total clearance of a compound and the average plasma concentration at steady state (Klaasen et al. 1986). The derived equation is (Klaasen et al. 1986):

$$ {k}_0=\frac{\mathrm{Clp}\times \mathrm{Cp}}{A}\kern0.5em $$

*k*_0_ is the daily intake of OTA (ng OTA/kg bw/day), *Clp* is the plasma clearance (ml/kg bw/day), *Cp* is the plasma concentration of OTA (ng/ml), and *A* is the bioavailability of OTA/fraction absorbed.

$$ {k}_0=\frac{\left(0.48\ \mathrm{ml}/\min \div 70\ \mathrm{kg}\times 1440\ \min \right)\times \mathrm{Cp}}{0.5} $$

$$ \to {k}_0=1.97\times \mathrm{Cp}\kern3.5em \left(\mathrm{Model}\ 1\right) $$

However, the human kinetic study by Studer-Rohr was re-published in 2000 and after re-calculation of the toxicokinetic data the renal clearance was calculated to be 0.1099 ml/min (Studer-Rohr et al. 2000). Imputing the updated value for renal clearance would yield a new coefficient for the model:

$$ {k}_0=4.52\times \mathrm{Cp}\kern3.5em \left(\mathrm{Model}\ 2\right) $$

Since most studies use model 1, we included both models in our calculations.

PDI for the population mean was calculated for the LB, MB and UB scenarios, as well as for the measured concentration of the highest exposed individual. PDIs were then compared with established health-based guidance values.

### Statistical analysis

All analyses were performed in STATA, version 12.1 and 14.1 (Stata Corporation, College Station, TX, USA). A *p* value < 0.05 was considered significant. Shapiro-Wilk’s test was used to investigate the normality of the data. Data on both mycotoxin concentrations and food consumption data, except for the intake of cereals and breads and the usual intakes of whole grain and dietary fibre, were nonnormally distributed. Data were treated accordingly to fit respective statistical test to deal with the nonnormality. We investigated the association between food consumption and mycotoxin concentrations (OTA, EnB and DON eqv) in median regression analysis. For the association between coffee and OTA, a censored regression analysis was used. Both regression models used bootstrapping over 1000 replications to produce robust estimates. The regression analyses were run both crude and adjusted for school year (categorical), sex (binary) and energy intake level (continuous). Association with OTA was also adjusted for household education (binary). To investigate the probability of exposure to 2’R-OTA in association with food consumption, a logistic regression model was run with 2’R-OTA concentration transformed into a binary variable, with values above LOD coded as 1 and values below LOD coded as 0. The logistic regression analysis was adjusted for same variables as in the other regression models with OTA. Differences in LB estimated concentrations of OTA and EnB in serum and DONeqv in urine were compared between the following groups: household education, living in different geographical regions (south, west, east and north), school year (5, 8 and 11) and sex, with two-sample Wilcoxon rank-sum (Mann-Whitney) test.

## Results

### Background characteristics

The background characteristics of the study participants, divided by school year, are presented in Table 1. The proportion of participating girls was the highest in the 11th school year. The mean reported intakes of cereals and breads were the highest in students in school year 8 as well was the energy intake. Background data and biomonitoring data were available for 1096 individuals. Of these, 1046 individuals had complete dietary recall data from all 3 days. Thus, for occurrence analyses, *n* = 1096, whilst for association analyses with long-term food intake with OTA, *n* = 1046.

### Comparison of method results for estimation of DON exposure

**Fig. 1:**
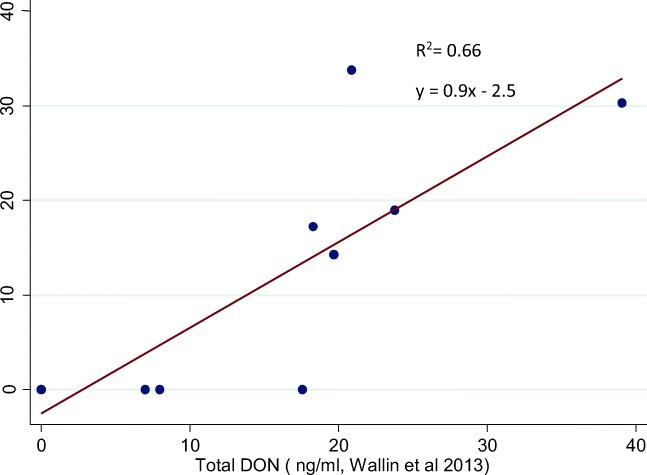
Correlation of total DON (ng/ml) in 10 urine samples collected in Riksmaten adults 2010–2011 (Wallin et al. 2013) measured by two different methods. The method used in Wallin et al. (2013) was immunoaffinity cleanup method (*x*-axis) and the second, the “dilute and shoot (DaS)” approach, the method used in the present study (*y*-axis)

The comparison of the 10 samples obtained in the study by Wallin et al. (2013) and the here determined concentrations revealed a good correlation and a linear relationship (*R*^2^ = 0.66) between the two methods (). The method used here has the advantage that sample clean-up is rather simple, rapid and fast. In combination with the short analysis time, a high sample throughput (*n* > 1000) is possible. However due to the simple sample clean-up, LOD/LOQ are for some compounds higher compared to other methods which use a time consuming sample clean-up either based on solid-phase extraction or immunoaffinity columns. For example, immunoaffinity cleanup, which was used in a multi mycotoxin analysis of the samples from the above mentioned study in adults (Mitropoulou et al. 2018; Wallin et al. 2015), resulted in more positive samples due to lower LOD/LOQ; however, the size of that study was limited. This can also be seen from the correlation analysis shown in Fig. 1. Five of the analysed samples using the DaS-approach correlate very well with the results obtained by the method used by Wallin et al. (2013). A few other samples were below LOD/LOQ as these values are higher for the DaS method.

### Occurrence of mycotoxin biomarkers in urine

The analysis of urine samples showed that DON and DON-15GlcA were the most frequently occurring mycotoxins, and 4.8 and 9.0% of the samples, respectively, were above the LOQ (Table 2). There were also a few positive samples with dihydrocitrinone (DH-CIT (1.5%)), HT-2-3-glucuronide (HT-2-3-GlcA (0.1%)) and OTA (0.1%). However, all those samples were below the LOQ. The concentrations of DON and DON-15GlcA in urine unadjusted for density or creatinine are also presented in Table 2. The concentrations of DON eqv (DON + DON-15GlcA) adjusted for density or creatinine are available in the Electronic Supplemental Material 3. The concentration of DON eqv (LB) in urine was not different between girls and boys, but there was a difference between the students in the different school years (*p* < 0.01), with the highest concentration in the youngest school year. Within each school year, there was no difference in concentration between the sexes. There were no regional differences in the concentration, but DON eqv was higher in adolescents with household education >12 years compared to ≤ 12 years (*p* = 0.03).

**Table 2:** Concentration of deoxynivalenol (DON) and DON-15-β-D-O-glucuronide (DON-15GlcA) in positive urine samples, unadjusted for density, divided by school year and sex

School year	Sex	Number	DON (ng/ml)				DON-15GlcA (ng/ml)			
			% > LOQ^1^	Mean^2^	Median	Max	% > LOQ^1^	Mean^2^	Median	Max
5	Female	165	6.7	8.4 ± 8.8	18.7	27.6	10.9	15.0 ± 8.5	12.4	38.3
	Male	166	1.8	27.1 ± 38.4	55.4	102.3	13.3	16.3 ± 14.3	12.1	58.7
8	Female	232	3.9	23.1 ± 23.0	32.7	82.4	6.0	19.8 ± 13.5	14.8	45.4
	Male	179	6.2	13.0 ± 18.2	46.6	46.6	7.9	21.5 ± 15.3	19.2	59.7
11	Female	219	4.6	16.8 ± 18.7	18.6	65.1	6.4	19.6 ± 10.0	16.3	40.6
	Male	135	3.0	10.5 ± 15.4	33.6	33.6	11.1	14.9 ± 6.7	13.3	29.4
	All	1096	4.8	16.0 ± 21.7	26.2	102.3	9.0	17.5 ± 11.8	13.6	59.7

Values between LOD and LOQ were replaced with ½ LOQ LOD for DON, 1.7 ng/ml, and for DON-15GlcA, 1.0 ng/ml

^1^LOQ (limit of quantitation) LOQ for DON, 5.6 ng/ml, and for DON-15GlcA, 3.3 ng/ml

^2^Mean is calculated in positive samples, excluding values < LOD (limit of detection)

### Occurrence of mycotoxin biomarkers in serum

OTA and EnB were frequently found in blood serum samples (Table 3). For OTA, 58.7% of the samples were above the LOQ, while all samples were above the LOD. Corresponding figures for EnB were 84.1% and 99.2%. Besides those toxins, 2’R-OTA was found in 8.3% of the samples of which 1.1% were above the LOQ (Table 3).

**Table 3:** Occurrence and concentration of ochratoxin A (OTA), 2’R-ochratoxin A (2’R-OTA) and enniatin B (EnB) in positive serum samples divided by school year and sex

School year	Sex	*n*	OTA				2’R-OTA				EnB			
			% > LOQ	Mean^1^ (ng/ml)	Median (ng/ml)	Max (ng/ml)	% > LOQ	Mean (ng/ml)	Median (ng/ml)	Max (ng/ml)	% > LOQ	Mean (ng/ml)	Median (ng/ml)	Max (ng/ml)
5	Female	165	60.6	0.059 ± 0.039	0.056	0.223	0	< ½ LOQ	< ½ LOQ	< LOQ	80.6	0.014 ± 0.013	0.010	0.093
	Male	166	69.3	0.059 ± 0.030	0.059	0.220	0	< ½ LOQ	< ½ LOQ	< LOQ	89.7	0.015 ± 0.013	0.012	0.113
8	Female	230	57.4	0.053 ± 0.030	0.053	0.178	0	< ½ LOQ	< ½ LOQ	< LOQ	80.0	0.013 ± 0.012	0.011	0.098
	Male	180	56.7	0.055 ± 0.034	0.056	0.237	1.1	0.036 ± 0.025	< ½ LOQ	0.098	61.7	0.013 ± 0.012	0.010	0.096
11	Female	219	58.0	0.053 ± 0.049	0.052	0.658	1.4	0.028 ± 0.012	< ½ LOQ	0.076	87.7	0.013 ± 0.009	0.010	0.060
	Male	136	49.3	0.050 ± 0.049	0.025	0.517	5.1	0.037 ± 0.027	< ½ LOQ	0.136	56.2	0.018 ± 0.013	0.014	0.080
	All	1096	58.7	0.055 ± 0.039	0.054	0.658	1.1	0.032 ± 0.020	< ½ LOQ	0.136	84.1	0.014 ± 0.012	0.011	0.113

Values between LOD and LOQ were replaced with ½ LOQ OTA LOQ, 0.05 ng/ml; LOD, 0.014 ng/ml; 2’R-OTA LOQ, 0.05 ng/ml; LOD, 0.014 ng/ml; EnB LOQ, 0.005 ng/ml; LOD, 0.0013 ng/ml

*n* number of participants, *LOQ* limit of quantitation

^1^Mean is calculated in positive samples, excluding values < LOD (limit of detection)

OTA serum concentration did not differ between the sexes but between the students from the different school years (*p* < 0.001). The concentration of OTA was the highest in students in the 5th school year. There were no differences between regions or household education levels in the concentration in OTA. 2’R-OTA serum concentration differed both between the sexes (higher in males, *p* = 0.03) and between the students from the different school years (p < 0.001). The 2’R-OTA serum concentration was higher in the 11th school year, compared to the 5th (*p* = 0.002) and 8th (*p* = 0.010) school year. Adolescents living in households with more than 12 years education, have lower mean 2’R-OTA serum levels, than adolescents with fewer years of household education (*p* = 0.009). There was no significant difference in coffee consumption between the educational level groups. The concentrations of EnB (LB) differed between the students from the different school years (*p* < 0.03) and between the sexes (*p* < 0.007), with boys having the highest concentrations. The concentration of EnB differed between the sexes within the 11th school year (*p* < 0.001). There were no differences between regions or household education levels in the concentration of EnB.

### Concentration of mycotoxin biomarkers in urine or serum and correlation to food consumption

#### DON

The number of consumers of barley (whole grain (*n* = 41), sifted (*n* = 20)) and corn (*n* = 12) were very low and therefore not investigated in the median regression models. The regression models revealed significant associations between DON eqv and intakes of oats, dietary fibre and whole grain. There was also a borderline significant association with whole grain rye and rice. All associations remained after adjustment for school year, sex and energy intake (Table 4).

**Table 4:** The direction (positive (+) or negative (−)) of the associations and *p* values from the crude regression analysis between foods, nutrients and mycotoxins detected in urine and serum

		DON equivalents in urine		Enniatin B in serum		OTA in serum	
	n		*p* value		*p* value		*p* value
Breads and cereal products, day 1	1096	*			0.002	–	0.46
Breads, day 1	1096	*		+	< 0.001	+	0.18
Oats, day 1	491	+	0.01	+	0.01	+	0.07
Whole grain rice, day 1	297	+	0.11	+	0.003	–	0.66
Rice, day 1	383	+	0.06	+	< 0.001	–	0.60
Rye, day 1	644	+	0.08	+	0.023	+	0.33
Whole grain wheat, day 1	414	–	0.27	+	< 0.001	+	0.33
Sifted wheat, day 1	1077	–	0.18	+	< 0.001	–	0.64
Wholegrain, day 1	972	+	0.004	+	0.17	–	0.13
Wholegrain, usual	1096	+	0.001	+	0.03	–	0.05
Dietary fibre, day 1	1096	+	0.009	+	< 0.001	+	0.03
Dietary fibre, usual	1096	+	0.004	+	< 0.001	+	0.13

Usual refers to the long-term intake based on intake from all 3 days of registration. The regression analysis was a median regression model, using bootstrapping over 1000 replications to produce robust estimates

*n* number of consumers, *DON* deoxinivalenol, *OTA* ochratoxin A

#### EnB

EnB was associated with all investigated foods and commodities and all associations remained after adjustment for school year, sex and energy intake, except the association to breads and cereal products.

#### OTA

Fibre intake (day 1) and oats (day 1) and were associated with higher OTA serum concentrations (Table 4). In the censored regression model, OTA concentration in serum was significantly related to the usual consumption of raisins (*p* = 0.04), coffee (*p* = 0.001) and oats (*p* = 0.02) as well as the consumption of peanuts (*p* = 0.03) on the day before the sampling.

The logistic regression analysis showed that there was an increased odds of detecting 2’R-OTA in serum with the consumption of the usual intake of coffee (odds ratio (OR) =1.77 (95% confidence interval (CI) 1.55; 2.02), dried peas (OR = 1.08 (95% CI 1.03; 1.13) and rice (OR = 1.02 (95% CI 1.003; 1.03). Coffee consumption the day previous to blood sampling was also strongly associated with 2’R-OTA detected in serum, as was consumption of fruit and nut mix, peanuts, dried peas and wheat bran (data not shown).

### Estimation of probable daily intake from urinary or serum biomarker concentration

The estimations of PDI of DON eqv (DON+ DON-15GlcA) were calculated using both density adjusted and creatinine adjusted data to enable comparisons with other studies (Table 5). Furthermore, for the same reason, both medians and means are presented even though, when estimating the daily intake, medians are preferred compared to the means due to the skewness of the data. Three different scenarios (LB, MB and UB) were estimated using the substitution method for the replacement of left censored values (EFSA 2010).

**Table 5:** Probable daily intakes (PDI, ng/kg bw) for total DON equivalents (DON+DON-15GlcA) calculated from urine samples adjusted for density or creatinine

School year	Sex	*n*	PDI DON eqv. (ng/kg bw) density adjusted				*n*^1^	PDI DON eqv (ng/kg bw) creatinine adjusted			
			LB mean ± SD (median)	MB mean ± SD (median)	UB mean ± SD (median)	Maximum		LB mean ± SD (median)	MB mean ± SD (median)	UB mean ± SD (median)	Maximum
5	Female	165	49 ± 160 (0)	91 ± 160 (40)	130 ± 170 (79)	1050	157	43 ± 180 (0)	74 ± 180 (26)	110 ± 180 (53)	1750
	Male	166	92 ± 490 (0)	131 ± 480 (38)	170 ± 480 (76)	4290	160	41 ± 200 (0)	76 ± 200 (29)	110 ± 210 (58)	2220
8	Female	232	44 ± 250 (0)	79 ± 250 (29)	1140 ± 240 (58)	1970	224	35 ± 170 (0)	67 ± 170 (27)	100 ± 180 (55)	1870
	Male	179	38 ± 190 (0)	68 ± 190 (26)	99 ± 190 (51)	1600	170	40 ± 180 (0)	74 ± 180 (28)	110 ± 190 (56)	1590
11	Female	219	59 ± 300 (0)	114 ± 300 (42)	170 ± 300 (85)	3340	210	42 ± 290 (0)	73 ± 290 (26)	100 ± 290 (53)	3009
	Male	135	33 ± 120 (0)	75 ± 120 (36)	120 ± 130 (71)	1040	123	94 ± 370 (0)	124 ± 360 (29)	160 ± 360 (57)	2570
Total	All	1096	53 ± 280 (0)	94 ± 280 (35)	130 ± 280 (69)	4290	1044	46 ± 240 (0)	78 ± 240 (28)	110 ± 240 (55)	3009

*eqv* equivalents, *n* number of participants, *LB* lower bound, *MB* middle bound, *UB* upper bound

^1^Samples with creatinine levels < 0.3 or > 3.0 mg/ml are deleted according to Cocker et al. (2011)

The PDI of DON eqv exceeded the TDI for DON (EFSA 2017) of 1 μg/kg bw in 1.6% (*n* = 18) of the participants (density adjusted data, *n* = 1096) and the maximum individual intake was 4.3 μg/kg bw which is about 50% of the acute reference dose (ARfD) of 8 μg/kg bw established by EFSA. Corresponding intake calculations using creatinine adjusted data (*n* = 1044) exceed TDI in 1.3% of the individuals and the maximum individual intake was 3.0 μg/kg bw. Furthermore, 39% of the individuals that exceeded the TDI were from county Västra Götaland, which is an area in the West of Sweden where DON contamination of cereals, especially in oats, are commonly higher than the rest of the country (Fredlund et al. 2013). The total number of participants from Västra Götaland corresponded to 20% of all participants.

The mean PDI of OTA in the study sample estimated for each of two toxicokinetic models and the LB, MB and UB scenarios are presented in Table 6. The mean PDI in the sample population ranged from 0.09 to 0.29 ng/kg bw/day. The lowest estimation was at the LB scenario calculated by model 1, which has the smallest coefficient of the models. The highest estimation for the sample population PDI was calculated using model 2, at the UB scenario. The highest measured serum OTA concentration in the sample population was estimated be equivalent of a PDI between 1.23 and 2.97 ng/kg bw/day which is about 10 times lower than the current TDI of 17.1 ng/kg bw established by EFSA (EFSA 2006). The EFSA opinion (EFSA 2006) is currently under full re-evaluation by EFSA.

**Table 6:** Probable daily intake (PDI) estimation of lower bound, middle bound and upper bound means based on OTA concentration in serum, calculated using two different models

	OTA PDI (ng/kg bw/day)			
	LB	MB	UB	Max
Model 1 (*k*_0_ = 1.97 × Cp) (Studer-Rohr 1995a)^1^	0.09	0.11	0.13	1.23
Model 2 (*k*_0_ = 4.52 × Cp) (Studer-Rohr et al. 2000)^1^	0.20	0.25	0.29	2.97

*OTA* ochratoxin A, *LB* lower bound, *MB* middle bound, *UB* upper bound, *Max* maximum intake, *Cp* plasma concentration, *k*_*0*_ mean daily intake

^1^Based on renal clearance

An attempt to roughly calculate the citrinin (CIT) exposure was also made using the recent preliminary data on CIT toxicokinetics (Ali et al. 2018). Using an UB scenario for DH-CIT and CIT concentration in urine (data not shown), the mean body weight (bw) of all participants (57.2 kg) and a urine volume of 1500 ml gave an UB mean PDI of 176 ng/kg bw which was below the level of no concern for nephrotoxicity at 200 ng/kg bw as proposed by EFSA (EFSA 2012).

## Discussion

The present study is unique due to the large study sample of adolescents (*n* = 1096) with paired data from the food consumption survey and the mycotoxin biomarker analysis. The Riksmaten adolescents survey introduced a new Web-based method for dietary assessment and questionnaires (Moraeus et al. 2018). The acquired data were useful in the current investigation of mycotoxin biomarkers and their association with food intake and other characteristics. Although there is an inherent difficulty to assess diet in a population due to misreporting and biases, we were able to detect significant associations between reported foods and mycotoxins levels in blood and urine. Further, the Web-based method, RiksmatenFlex Diet, has been shown valid for use in an adolescent population (Lindroos et al. 2019).

The urinary concentration of total DON was generally low among Swedish adolescents (mean of 16.0 ± 21.7 ng/ml, *n* = 1096), except for a few individuals for which a higher concentration in urine was detected (maximum 102.3 ng/ml). Corresponding figures for adolescents in the UK were 27.0 ng/ml and a maximum at 104.3 ng/ml (Papageorgiou et al. 2018). The data from the UK were also published in an EFSA supporting document (Brera et al. 2015), which also included data from adolescents in Italy and Norway. The concentration of total DON in these two countries was much lower than in the UK. The number of participants from each country was low, around 40, which could have influenced the urinary concentration besides the contamination level in food.

In the present study, the mean serum OTA concentration was 0.055 ng/ml. In the majority of previous biomonitoring studies on serum OTA, the study populations have been adults. To the authors’ knowledge, the only other blood biomonitoring study including children aged 6–18 years (*n* = 7–14) was conducted in two separate regions of Turkey during two separate seasons (Erkekoglu et al. 2010). Mean OTA concentrations were 0.285 and 0.094 ng/ml in each region during winter and 0.877 and 0.161 ng/ml in the summer. In the present study, no regional differences in OTA exposure were detected, and no investigation was done on seasonal difference. In comparison, Swedish adolescents have lower OTA exposure than the Turkish adolescents.

Previous Swedish biomonitoring studies have revealed significantly higher OTA blood concentration in residents on the island of Gotland compared to residents of two Swedish and Norwegian mainland cities (Breitholtz et al. 1991). It was hypothesised that the difference was due to that the island population was more dependent on local food products combined with local food storage practices. In the present sample population, such a difference could not be detected. In fact, mean OTA exposure in the present investigation is more comparable to those previously measured in mainland residents, than at Gotland. Developments in the last 20–30 years leading to better food storage practices and accessibility for trade between regions in Sweden could be a possible explanation for this.

There were no regional differences detected concerning neither DON nor EnB concentrations in the present investigation. However, 39% of the individuals having a PDI exceeding the health-based guidance value (HBGV) for DON (2017) were living in Västra Götaland county. This may be connected with high levels of DON in grain, particularly in oats grown in this area of Sweden (Fredlund et al. 2013). Furthermore, pigs from farms in the same county have significantly higher urinary concentration of DON than pigs from two other Swedish major grain producing areas (Gambacorta et al. 2019).

Enniatins are very common in Swedish wheat and oats (Fredlund et al. 2013), which explains the high percentage of positive serum samples in this study. Depending on the lack of human toxicokinetics data and a HBGV, the serum concentration was difficult to evaluate. However, the concentrations were in line with other studies (Viegas et al. 2018). Toxicity and toxicokinetics in other species have recently been reviewed by Fraeyman (Fraeyman et al. 2017) and as an EFSA supporting document (Maranghi et al. 2018), which hopefully makes it possible to risk assess enniatins in the near future.

With an average content of 2.3 ng OTA per gram, raisins was found to be the most contaminated food item in the Canadian Total Diet Study (Tam et al. 2011). In our study, reported intake of raisins was particularly associated with higher OTA exposure. However, this must be put in relation to portion sizes. Although grains and grain-based products have lower average OTA contamination, the average consumption is several times higher than raisin consumption (Ostry et al. 2015) and as reported in the present study (Table 1).

Boys in the 11th school year had the highest concentrations of serum 2’R-OTA. The mean 2’R-OTA serum concentration was 0.032 ng/ml and the highest concentration 0.136 ng/ml. There are a limited number of biomonitoring studies measuring 2’R-OTA and, to our knowledge, no other studies with an adolescent study population. Viegas et al. (2018) detected 2’R-OTA in the blood of 81% of waste management workers (*n* = 42). The mean concentration was 0.334 ng/ml and the maximum was 0.627 ng/ml. In another study, the mean 2’R-OTA concentration was 0.11 ng/ml and maximum 0.414 ng/ml (Cramer et al. 2015). Considering this, Swedish adolescents have lower 2’R-OTA concentrations than other tested populations. Until now, coffee is the only food item where 2’R-OTA has been found in large quantities (Cramer et al. 2015; Studer-Rohr et al. 1995b; Sueck et al. 2019a), but traces of 2’R-OTA have recently been found in coffee surrogates and dark rye bread (Sueck et al. 2019b). Coffee consumption is more common among adults than adolescents, which would explain why our study population sample has lower 2’R-OTA concentrations than other groups. Interestingly, we could see associations between 2’R-OTA exposure and dried peas and rice, in addition to the coffee consumption. Although the associations are modest, the findings suggest that other food items than coffee may be sources for 2’R-OTA contamination. Furthermore, cohorts of children and adolescents may be useful to detect alternative sources for 2’R-OTA. Since there are no HBGV and few toxicity studies, it is not possible to make a risk assessment of 2’R-OTA.

DON and EnB associations were only tested against cereal based food groups, whole grain and dietary fibre. EnB had a strong association with most tested cereal food group and to dietary fibre, which is most likely related to its abundant occurrence in Swedish grain described above. Further, DON concentration is the highest in the bran fraction of cereals (L’vova et al. 1998; Vidal et al. 2013). However, its association to rice was negative but strong, which indicates that rice was not as contaminated as the other cereals. If rice consumption was increased in the diet, the EnB serum concentration would consequently be reduced. Low contamination of enniatins in rice is supported by other studies (Decleer et al. 2016; Nazari et al. 2015). In addition, DON concentration in urine were higher in the younger adolescents and in those with household education > 12 years compared to ≤ 12 years (*p* = 0.03). It is likely that parents with higher education are more aware of the advice to increase wholegrain in the diet, partly explaining the results. It was not a part of this study to risk benefit assess the consumption of wholegrain, but it is highly recommended to do so to reveal or exclude risk in comparison with benefits associated with wholegrain consumption.

Considering LB, MB and UB, as well as the two different models to calculate the PDI of OTA, the estimated mean PDI falls within the range 0.09–0.29 ng/kg bw/day. In 2006, EFSA proposed a tolerably weekly intake (TWI) for OTA to be 120 ng/kg bw/week which corresponds to a TDI of approximately 17 ng/kg bw/day (EFSA 2006). For the majority of our sample population, OTA exposure is well below existing TDI. Although a few subjects have 10 times higher OTA exposure, even the most cautious model calculates a PDI well below the TDI set by EFSA. The margin of exposure for our population is much narrower when comparing to for example Canadian guidance-values (Kuiper-Goodman et al. 2010). Assuming OTA to be a non-threshold carcinogen, Kuiper-Goodman (Kuiper-Goodman et al. 2010) calculated the negligible cancer risk intake level OTA to be 4 ng/kg bw/day and proposed using this value as a HBGV.

Intra-individual variations in absorption distribution metabolism excretion (ADME)-related functions as well the long half-life of OTA, leads to high uncertainty in calculating the probable intake from biomarker measurements in blood samples (Ali et al. 2017). As of yet, methods for intake estimation of OTA lack verification from lager study groups, as the values were determined from one single individual (Studer-Rohr 1995a; Studer-Rohr et al. 1995b; Studer-Rohr et al. 2000). Nonetheless, several studies propose intake estimations based on the available toxicokinetic data. Unfortunately, there are several different calculations used (Coronel et al. 2010). Moreover, several studies have published versions of intake calculations that are incorrect due to misinterpretations of the human toxicokinetic data (Ali et al. 2017). OTA intake estimation from urine has also been successfully done (Gilbert et al. Gilbert and Brereton 2001; Munoz et al. 2014). However, due to the low urinary excretion rate, the limited sensitivity of the applied dilute and shoot approach as well as the overall low exposure to OTA in the cohort, only 0.1% of the urine samples were positive for OTA. Consequently, calculations were only made for serum samples and evaluation of the results should focus on comparison with previous studies, rather than accurate determination of individual OTA exposure.

The PDI of DON among Swedish adolescents was in the same range as found in the previous national survey, Riksmaten adults 2010–2011 (Mitropoulou et al. 2018). There are, as mentioned earlier, several uncertainties connected with estimation of PDI from biomarker data. In the case of DON having a rapid elimination, around 70% are excreted within 24 h, and the time laps between meals with DON-contaminated food and the urine spot sampling will substantially influence the urinary concentration found. However, it is not feasible to collect 24-h urine samples in a survey like this. Yet, we did find associations between the DON concentration and the well-known food sources of DON, which indicates that the sampling procedure is probably good enough. The following conclusions can be drawn from the study:

• Urinary DON concentration is generally low but approximately 2% of the participants had a PDI above the group TDI for DON.

• This study showed that OTA is a ubiquitous food contaminant among Swedish adolescents based on serum data.

• Association to cereals were shown for all mycotoxins (DON, OTA and EnB). OTA was also associated with for example raisins and coffee.

• The study also confirms the correlation between coffee consumption and 2’R-OTA exposure and suggests that 2’R-OTA exposure could be correlated to socioeconomic factors.

## Electronic supplementary material

ESM 1 (DOCX 15 kb)

ESM 2 (DOCX 29 kb)

ESM 3 (DOCX 17 kb)

## References

[CR1] Ali N, Blaszkewicz M, Degen GH (2016). Assessment of deoxynivalenol exposure among Bangladeshi and German adults by a biomarker-based approach. Toxicol Lett.

[CR2] Ali N, Munoz K, Degen GH (2017). Ochratoxin A and its metabolites in urines of German adults-An assessment of variables in biomarker analysis. Toxicol Lett.

[CR3] Ali N, Hossain K, Degen GH (2018). Blood plasma biomarkers of citrinin and ochratoxin A exposure in young adults in Bangladesh. Mycotoxin Res.

[CR4] Breitholtz A, Olsen M, Dahlback A, Hult K (1991). Plasma ochratoxin A levels in three Swedish populations surveyed using an ion-pair HPLC technique. Food Addit Contam.

[CR5] Brera C et al. (2015) Experimental study of deoxynivalenol biomarkers in urine EFSA Supporting Publications 12 doi:10.2903/sp.efsa.2015.EN-818

[CR6] Chen L, Yu M, Wu Q, Peng Z, Wang D, Kuca K, Yao P, Yan H, Nussler AK, Liu L, Yang W (2017). Gender and geographical variability in the exposure pattern and metabolism of deoxynivalenol in humans: a review. J Appl Toxicol.

[CR7] Cocker J, Mason HJ, Warren ND, Cotton RJ (2011). Creatinine adjustment of biological monitoring results. Occup Med (Lond).

[CR8] Coronel MB, Sanchis V, Ramos AJ, Marin S (2010). Review. Ochratoxin A: presence in human plasma and intake estimation. Food Sci Technol Int.

[CR9] Cramer B, Osteresch B, Munoz KA, Hillmann H, Sibrowski W, Humpf HU (2015). Biomonitoring using dried blood spots: detection of ochratoxin A and its degradation product 2'R-ochratoxin A in blood from coffee drinkers. Mol Nutr Food Res.

[CR10] Decleer M, Rajkovic A, Sas B, Madder A, De Saeger S (2016). Development and validation of ultra-high-performance liquid chromatography-tandem mass spectrometry methods for the simultaneous determination of beauvericin, enniatins (A, A1, B, B1) and cereulide in maize, wheat, pasta and rice. J Chromatogr A.

[CR11] Degen GH (2011). Tools for investigating workplace-related risks from mycotoxin exposure. World Mycotoxin J.

[CR12] Dietrich DR, Heussner AH, O'Brien E (2005). Ochratoxin A: comparative pharmacokinetics and toxicological implications (experimental and domestic animals and humans). Food Addit Contam.

[CR13] Duarte SC, Pena A, Lino CM (2011). Human ochratoxin a biomarkers--from exposure to effect. Crit Rev Toxicol.

[CR14] EFSA (2006). Scientific opinion on the risks related to ochratoxin A in food. EFSA J.

[CR15] EFSA (2010). Management of left-censored data in dietary exposure assessment of chemical substances. EFSA J.

[CR16] EFSA (2012). Scientific opinion on the risks for public and animal health related to the presence of citrinin in food and feed. EFSA J.

[CR17] EFSA (2016). Appropriateness to set a group health-based guidance value for zearalenone and its modified forms. EFSA J.

[CR18] EFSA (2017). Scientific opinion on the risks to human and animal health related to the presence of deoxynivalenol and its acetylated and modified forms in food and feed Deoxynivalenol, 3-acetyl-deoxynivalenol, 15-acetyl-deoxynivalenol. EFSA J.

[CR19] Erkekoglu P, Sabuncuoglu S, Aydin S, Sahin G, Giray B (2010). Determination of seasonal variations in serum ochratoxin A levels in healthy population living in some regions of Turkey by enzyme-linked immunosorbent assay. Toxicon.

[CR20] Faeste CK, Ivanova L, Sayyari A, Hansen U, Sivertsen T, Uhlig S (2018). Prediction of deoxynivalenol toxicokinetics in humans by in vitro-to-in vivo extrapolation and allometric scaling of in vivo animal data. Arch Toxicol.

[CR21] Fraeyman S, Croubels S, Devreese M, Antonissen G (2017) Emerging Fusarium and Alternaria mycotoxins: occurrence, toxicity and toxicokinetics. Toxins (Basel) 9. 10.3390/toxins9070228 10.3390/toxins9070228 PMC5535175 28718805

[CR22] Fredlund E, Gidlund A, Sulyok M, Borjesson T, Krska R, Olsen M, Lindblad M (2013). Deoxynivalenol and other selected Fusarium toxins in Swedish oats--occurrence and correlation to specific Fusarium species. Int J Food Microbiol.

[CR23] Gambacorta L, Olsen M, Solfrizzo M (2019) Pig urinary concentration of mycotoxins and metabolites reflects regional differences, mycotoxin intake and feed contaminations. Toxins (Basel) 11. 10.3390/toxins11070378 10.3390/toxins11070378 PMC6669694 31262000

[CR24] Gerding J, Ali N, Schwartzbord J, Cramer B, Brown DL, Degen GH, Humpf HU (2015). A comparative study of the human urinary mycotoxin excretion patterns in Bangladesh, Germany, and Haiti using a rapid and sensitive LC-MS/MS approach. Mycotoxin Res.

[CR25] Gilbert J, Brereton P, MacDonald S (2001) Assessment of dietary exposure to ochratoxin A in the UK using a duplicate diet approach and analysis of urine and plasma samples. Food Addit Contam 18:1088–1093 doi:10.1080/02652030110070030 10.1080/02652030110070030 11761119

[CR26] Gratz SW, Duncan G, Richardson AJ (2013). The human fecal microbiota metabolizes deoxynivalenol and deoxynivalenol-3-glucoside and may be responsible for urinary deepoxy-deoxynivalenol. Appl Environ Microbiol.

[CR27] Heyndrickx E, Sioen I, Huybrechts B, Callebaut A, De Henauw S, De Saeger S (2015). Human biomonitoring of multiple mycotoxins in the Belgian population: results of the BIOMYCO study. Environ Int.

[CR28] Klaasen CD, Amdur M, Doull OJ (1986). Casarett and Doull's toxicology: the basic science of poisons.

[CR29] Kuiper-Goodman T, Hilts C, Billiard SM, Kiparissis Y, Richard ID, Hayward S (2010). Health risk assessment of ochratoxin A for all age-sex strata in a market economy. Food Addit Contam Part A Chem Anal Control Expo Risk Assess.

[CR30] L’vova LS, Kizlenko OI, Shul’gina AP, Omel’chenko MD, Bystryakova ZK (1998). Distribution of deoxynivalenol in products of processing Fusarium-affected soft and hard wheats and barley. Appl Biochem Biotechnol.

[CR31] Lindroos AK, Petrelius Sipinen J, Axelsson C, Nyberg G, Landberg R, Leanderson P, Arnemo M, Warensjo Lemming E (2019). Use of a web-based dietary assessment tool (RiksmatenFlex) in Swedish adolescents: comparison and validation study. J Med Internet Res.

[CR32] Maranghi F et al. (2018) In vivo toxicity and genotoxicity of beauvericin and enniatins. Combined approach to study in vivo toxicity and genotoxicity of mycotoxins beauvericin (BEA) and enniatin B (ENNB). EFSA supporting publications 15 doi:10.2903/sp.efsa.2018.EN-1406

[CR33] Märtlbauer E, Usleber E, Dietrich R, Schneider E (2009). Ochratoxin A in human blood serum – retrospective long-term data. Mycotoxin Research.

[CR34] Mazzachi BC, Peake MJ, Ehrhardt V (2000). Reference range and method comparison studies for enzymatic and Jaffe creatinine assays in plasma and serum and early morning urine. Clin Lab.

[CR35] Mitropoulou A, Gambacorta L, Warensjö Lemming E, Solfrizzo M, Olsen M (2018). Extended evaluation of urinary multi-biomarker analyses of mycotoxins in Swedish adults and children. World Mycotoxin J.

[CR36] Moraeus L, Lemming EW, Hursti UK, Arnemo M, Sipinen JP, Lindroos AK (2018) Riksmaten Adolescents 2016-17: a national dietary survey in Sweden - design, methods, and participation. Food Nutr Res 62. 10.29219/fnr.v62.1381 10.29219/fnr.v62.1381 PMC6116384 30186087

[CR37] Munoz K, Blaszkewicz M, Campos V, Vega M, Degen GH (2014). Exposure of infants to ochratoxin A with breast milk. Arch Toxicol.

[CR38] Nazari F, Sulyok M, Kobarfard F, Yazdanpanah H, Krska R (2015). Evaluation of emerging Fusarium mycotoxins beauvericin, Enniatins, Fusaproliferin and Moniliformin in domestic rice in Iran. Iran J Pharm Res.

[CR39] O'Brien E, Heussner AH, Dietrich DR (2001) Species-, sex-, and cell type-specific effects of ochratoxin A and B. Toxicol Sci 63:–256, 264. 10.1093/toxsci/63.2.256 10.1093/toxsci/63.2.256 11568369

[CR40] Osteresch B, Viegas S, Cramer B, Humpf HU (2017). Multi-mycotoxin analysis using dried blood spots and dried serum spots. Anal Bioanal Chem.

[CR41] Ostry V, Malir F, Dofkova M, Skarkova J, Pfohl-Leszkowicz A, Ruprich J (2015). Ochratoxin A dietary exposure of ten population groups in the Czech Republic: comparison with data over the world. Toxins (Basel).

[CR42] Pacin AM, Ciancio Bovier EV, Motta E, Resnik SL, Villa D, Olsen M (2008). Survey of Argentinean human plasma for ochratoxin A. Food Addit Contam Part A Chem Anal Control Expo Risk Assess.

[CR43] Papageorgiou M et al (2018) Assessment of urinary deoxynivalenol biomarkers in UK children and adolescents. Toxins (Basel):10. 10.3390/toxins10020050 10.3390/toxins10020050 PMC5848151 29360781

[CR44] Smith KW, Braun JM, Williams PL, Ehrlich S, Correia KF, Calafat AM, Ye X, Ford J, Keller M, Meeker JD, Hauser R (2012) Predictors and variability of urinary paraben concentrations in men and women, including before and during pregnancy. Environ Health Perspect, 120:1538–1543. 10.1289/ehp.1104614 10.1289/ehp.1104614 PMC3556607 22721761

[CR45] Solfrizzo M, Gambacorta L, Visconti A (2014). Assessment of multi-mycotoxin exposure in southern Italy by urinary multi-biomarker determination. Toxins (Basel).

[CR46] Soto JB, Ruiz MJ, Manyes L, Juan-Garcia A (2016). Blood, breast milk and urine: potential biomarkers of exposure and estimated daily intake of ochratoxin A: a review. Food Addit Contam Part A Chem Anal Control Expo Risk Assess.

[CR47] Studer-Rohr I (1995). Ochratoxin A in humans: exposure, kinetics and risk assessment.

[CR48] Studer-Rohr I, Dietrich DR, Schlatter J, Schlatter C (1995). The occurrence of ochratoxin A in coffee. Food Chem Toxicol.

[CR49] Studer-Rohr I, Schlatter J, Dietrich DR (2000). Kinetic parameters and intraindividual fluctuations of ochratoxin A plasma levels in humans. Arch Toxicol.

[CR50] Sueck F, Cramer B, Czeschinski P, Humpf HU (2019). Human study on the kinetics of 2'R-ochratoxin a in the blood of coffee drinkers. Mol Nutr Food Res.

[CR51] Sueck F, Hemp V, Specht J, Torres O, Cramer B, Humpf H-U (2019). Occurrence of the ochratoxin a degradation product 2′R-ochratoxin a in coffee and other food: an update. Toxins.

[CR52] Suwazono Y, Akesson A, Alfven T, Jarup L, Vahter M (2005). Creatinine versus specific gravity-adjusted urinary cadmium concentrations. Biomarkers.

[CR53] Tam J, Pantazopoulos P, Scott PM, Moisey J, Dabeka RW, Richard ID (2011). Application of isotope dilution mass spectrometry: determination of ochratoxin A in the Canadian Total Diet Study. Food Addit Contam Part A Chem Anal Control Expo Risk Assess.

[CR54] Turner PC, White KL, Burley VJ, Hopton RP, Rajendram A, Fisher J, Cade JE, Wild CP (2010). A comparison of deoxynivalenol intake and urinary deoxynivalenol in UK adults. Biomarkers.

[CR55] Vidal A, Marin S, Ramos AJ, Cano-Sancho G, Sanchis V (2013). Determination of aflatoxins, deoxynivalenol, ochratoxin A and zearalenone in wheat and oat based bran supplements sold in the Spanish market. Food Chem Toxicol.

[CR56] Vidal A, Claeys L, Mengelers M, Vanhoorne V, Vervaet C, Huybrechts B, De Saeger S, De Boevre M (2018). Humans significantly metabolize and excrete the mycotoxin deoxynivalenol and its modified form deoxynivalenol-3-glucoside within 24 hours. Sci Rep.

[CR57] Viegas S, Osteresch B, Almeida A, Cramer B, Humpf HU, Viegas C (2018). Enniatin B and ochratoxin A in the blood serum of workers from the waste management setting. Mycotoxin Res.

[CR58] Viegas S, Assuncao R, Martins C, Nunes C, Osteresch B, Twaruzek M, Kosicki R, Grajewski J, Ribeiro E, Viegas C (2019) Occupational exposure to mycotoxins in swine production: environmental and biological monitoring approaches. Toxins (Basel):11. 10.3390/toxins11020078 10.3390/toxins11020078 PMC6410041 30717100

[CR59] Wallin S, Hardie LJ, Kotova N, Lemming EW, Nälsén C, Ridefelt P, Turner PC, White KLM, Olsen M (2013). Biomonitoring study of deoxynivalenol exposure and association with typical cereal consumption in Swedish adults. World Mycotoxin J.

[CR60] Wallin S, Gambacorta L, Kotova N, Lemming EW, Nalsen C, Solfrizzo M, Olsen M (2015). Biomonitoring of concurrent mycotoxin exposure among adults in Sweden through urinary multi-biomarker analysis. Food Chem Toxicol.

[CR61] Warth B, Sulyok M, Fruhmann P, Berthiller F, Schuhmacher R, Hametner C, Adam G, Frohlich J, Krska R (2012). Assessment of human deoxynivalenol exposure using an LC-MS/MS based biomarker method. Toxicol Lett.

