# Supplementary material for: Mycotoxins in blood and urine of Swedish adolescents—possible associations to food intake and other background characteristics
Source: Mycotoxin Res. 2019 Dec 14;36(2):193–206. doi: 10.1007/s12550-019-00381-9 (PMC7182616; doi:10.1007/s12550-019-00381-9)
Supplement: Supplementary file 1 — (DOCX 15 kb) [file 12550_2019_381_MOESM1_ESM.docx]

**Electronic Supplemental Material 1**. Limits of detection (LOD), Limits of quantitation (LOQ) and working range for the matrix-matched calibrations of the DaS method for urine analysis.

| Analyte | LOD [ng/mL]^a^ | LOQ [ng/mL]^a^ | Working range [ng/mL] |
| --- | --- | --- | --- |
| AFB1 | 0.01 (0.1) | 0.03 (0.3) | 0.02–4 |
| AFB2 | 0.01 (0.1) | 0.03 (0.3) | 0.005-1 |
| AFG1 | 0.02 (0.2) | 0.08 (0.8) | 0.02–4 |
| AFG2 | 0.05 (0.5) | 0.17 (1.7) | 0.005-1 |
| AFM1 | 0.006 (0.06) | 0.020 (0.20) | 0.025–5 |
| ALT | 0.3 (3) | 1.0 (10) | 0.25–50 |
| AME | 0.03 (0.3) | 0.10 (1.0) | 0.045–9 |
| AOH | 0.15 (1.5) | 0.50 (5.0) | 0.125–25 |
| BEA | 0.03 (0.3) | 0.10 (1.0) | 0.0125–2.5 |
| CIT | 0.25 (2.5) | 0.83 (8.3) | 0.25–50 |
| DH-CIT | 0.02 (0.2) | 0.07 (0.7) | 0.04–8 |
| DOM-1 | 0.60 (6.0) | 1.98 (19.8) | 0.5–100 |
| DON | 0.17 (1.7) | 0.56 (5.6) | 0.5–100 |
| DON-GlcA | 0.10 (1.0) | 0.33 (3.3) | 0.3–60 |
| EnA | 0.005 (0.05) | 0.017 (0.17) | 0.0035–0.7 |
| EnA1 | 0.005 (0.05) | 0.017 (0.17) | 0.0035–0.7 |
| EnB | 0.001 (0.01) | 0.003 (0.03) | 0.0015–0.3 |
| EnB1 | 0.005 (0.05) | 0.017 (0.17) | 0.0035–0.7 |
| FB1 | 0.3 (3) | 1.0 (10) | 0.125–25 |
| 10-OH-OTA | 0.01 (0.1) | 0.03 (0.3) | 0.0075–1.5 |
| HT-2 | 1.5 (15) | 5.0 (50) | 0.5–100 |
| HT-2-3-GlcA | 0.04 (0.4) | 0.13 (1.3) | 0.1–20 |
| HT-2-4-GlcA | 0.04 (0.4) | 0.13 (1.3) | 0.1–20 |
| NIV | 0.50 (5.0) | 1.65 (16.5) | 0.5–100 |
| OTA | 0.001 (0.01) | 0.003 (0.03) | 0.003–0.6 |
| 2’ROTA | 0.005 (0.05) | 0.017 (0.17) | 0.01–2 |
| OTα | 0.04 (0.4) | 0.13 (1.3) | 0.03–6 |
| T-2 | 0.02 (0.2) | 0.07 (0.7) | 0.02–4 |
| ZAN | 0.10 (1.0) | 0.33 (3.3) | 0.075–15 |
| ZEN | 0.03 (0.3) | 0.10 (1.0) | 0.04–15 |
| ZEN-14-GlcA | 0.50 (5) | 1.65 (16.5) | 0.5–100 |
| α-ZEL | 0.05 (0.5) | 0.17 (1.7) | 0.05–10 |
| β-ZEL | 0.06 (0.6) | 0.20 (2.0) | 0.075–15 |
| α-ZEL-GlcA | 1.5 (15) | 5.0 (50) | 0.5–100 |
| β-ZEL-GlcA | 0.75 (7.5) | 2.48 (24.8) | 0.5–100 |

Limit of detection (LOD) and limit of quantitation (LOQ) were determined at a S/N ratio of 3 (LOD) and 10 (LOQ). ^a^ Values in parentheses are LOD/LOQ in urine, considering the 1:10 dilution of the urine sample.
