# Supplementary material for: Mycotoxins in blood and urine of Swedish adolescents—possible associations to food intake and other background characteristics
Source: Mycotoxin Res. 2019 Dec 14;36(2):193–206. doi: 10.1007/s12550-019-00381-9 (PMC7182616; doi:10.1007/s12550-019-00381-9)
Supplement: Supplementary file 2 — (DOCX 29 kb) [file 12550_2019_381_MOESM2_ESM.docx]

**Electronic Supplemental Material 2.**

Chemicals and reagents

Acetonitrile (ACN) in HPLC-MS quality was obtained from Thermo Fisher Scientific (Schwerte, Germany) and acetone of LC grade purity from VWR (Darmstadt, Germany). Formic acid (FA) and acetic acid (AA) were from Merck (Darmstadt, Germany). ASTM Type 1 water was prepared using a Purelab Flex 2 system (Veolia Water Technologies, Celle, Germany). Whatman 903 protein saver cards™ for preparation of serum samples were purchased from Sigma-Aldrich (Taufkirchen, Germany). The sources for mycotoxin standards can be found in ([Gerding et al. 2015](#_ENREF_3); [Osteresch et al. 2017](#_ENREF_4)). Stock solutions were prepared at 10 or 20 µg/mL in ACN or ACN/H_2_O and stored at -18 °C. For urine analysis, two working solutions were prepared at 10- and 100-fold concentration of the highest calibration point, respectively. For serum samples, two working solutions at 10-fold concentration of the highest calibration point were prepared and stored at -18°C.

Sample preparation

*Urine samples*

Analysis of urine samples was performed according to Gerding et al. (2015), slightly modified by integration of further analytes. In total 35 mycotoxins and mycotoxin metabolites were measured in urine (AFB_1_, AFB_2_, AFG_1_, AFG_2_, AFM_1_, ALT, AME, AOH, BEA, CIT, DH-CIT, DOM-1, DON, DON-GlcA, EnA, EnA_1_, EnB, EnB_1_, FB_1_, 10-OH-OTA, HT-2, HT-2-3-GlcA, HT-2-4-GlcA, NIV, OTA, 2’R-OTA, OTα, T-2, ZAN, ZEN, ZEN-14-GlcA, α-ZEL, β-ZEL, α-ZEL-GlcA, β-ZEL-GlcA). Limits of detection (LOD), limits of quantitation (LOQ) and working range are displayed in the electronic supplemental material 1. For analysis urine samples were allowed to reach room temperature, homogenized vigorously and centrifuged for 5 min at 15000*g*. An aliquot of the supernatant (11.1 µL) was diluted with 100 µL H_2_O/ACN/FA (95/5/0.1, v/v/v) and subjected to HPLC-MS/MS analysis. For quantitation a matrix-matched calibration was used by spiking a blank urine sample with aliquots of the working solutions. The calibration curves consisted of eight calibration points and covered a working range of two magnitudes for each analyte. To assure method performance, linearity of the calibration curves for the detected analytes was confirmed by coefficients of correlation (R^2^) of 0.999 to 0.981. Detailed validation parameters are described in Gerding et al ([Gerding et al. 2015](#_ENREF_3)). As for DON-15-GlcA no reference standard was available, a standard solution of DON-3-GlcA was used for quantitation. The results were corrected by factor 1.88 due to the higher MS response of MRM transition m/z 471.1 → 265.2, which was used for quantitation, according to Warth et al. ([Warth et al. 2012](#_ENREF_7)). Due to the high number of samples measurements were performed in single analysis and a blank urine sample spiked with all analytes at medium concentration level was used for quality control in each batch of twenty samples. Samples positive for an analyte were prepared and analyzed in duplicate. To assure method performance 10 additional human urine samples were analysed for DON. The urine samples have previously been analysed within a biomonitoring study of DON exposure in Swedish adults ([Wallin et al. 2013](#_ENREF_6)).

Spiked blank urine samples were used to determine trueness and precision for the detected analytes. Trueness was expressed as percentage recovery of the spiked amount. To evaluate precision, relative standard deviation (RSD) during intraday and interday repeatability was determined. The following values for recovery, intraday repeatability and interday repeatability were determined: DH-CIT 53.7%/8.3%/8.0%, DON 81.6%/26.0%/18.8%, DON-3-GlcA 70.2%/22.6%/19.5%, HT-2-3-GlcA 85.2%/24.0%/27.3% and OTA 51.1%/10.9%/9.0%. Recovery was assessed as sufficient according to the EU commission decision 2002/657/EC ([European Commission 2002](#_ENREF_2)) concerning the performance of analytical methods and the interpretation of results. As decision 2002/657/EC provides no precise recommendations for reproducibility for the mass fraction of interest, the US Food & Drug Administration method acceptability criteria ([US Food & Drug Administration 2015](#_ENREF_5)) were considered to be appropriate. The determined values for intraday and interday precision were in accordance with therein indicated criteria.

*Blood serum samples*

Determination of biomarkers in blood serum (AFB_1_, AFB_2_, AFG_1_, AFG_2_, AFM_1_, ALT, AME, AOH, BEA, CIT, DH-CIT, DON, DON-GlcA, EnA, EnA_1_, EnB, EnB_1_, FB_1_, 10-OH-OTA, HT-2, HT-2-4-GlcA, OTA, 2’R-OTA, OTα,T-2, ZAN, ZEN) was carried out according to Osteresch et al. ([Osteresch et al. 2017](#_ENREF_4)). Obtained limits of detection were in a range of about 0.001 ng/mL to 1.3 ng/mL ([Osteresch et al. 2017](#_ENREF_4)). For analysis, serum was allowed to reach room temperature, homogenized vigorously and 100 µL of serum were pipetted on Whatman 903 protein saver cards™. After drying overnight at room temperature, the dried serum spots (DSS) were cut out and transferred into 2-mL safe-lock tubes followed by extraction with 1 mL of H_2_O/ACN/acetone (30/35/35, v/v/v) for 30 min under sonication. An aliquot of 600 µL was transferred into a new 2-mL safe-lock tube and evaporated to dryness at 45 °C under reduced pressure. The residues were reconstituted with 100 µL of H_2_O/ACN/AA (95/5/0.1, v/v/v) and centrifuged for 5 min at 15000*g* prior to HPLC-MS/MS analysis ([Osteresch et al. 2017](#_ENREF_4)). A matrix-matched calibration with dried serum spot extract (from a German serum sample) spiked with standard solutions was used for quantitation. Coefficients of correlation (R^2^) for the matrix-matched calibrations were calculated to be 0.999 to 0.990. The serum sample contained low concentrations of EnB, OTA and 2’R-OTA, as a blank matrix was not available. Single analysis was applied for all serum samples and a serum sample naturally contaminated with EnB, OTA and 2’R-OTA was analysed as quality control in each batch of twenty samples. Intraday repeatability and interday repeatability were determined to be the following values: EnB 19.0%/24.8%, OTA 14.0%/18.3% and 2’R-OTA 17.3%/24.8%. The values were in compliance with the method acceptable criteria of the FDA guideline as previously described. Recovery rates for serum analysis could not be calculated, because no blank matrix was available

Analysis

Analysis was carried out on an Agilent 1260 Infinity HPLC system coupled to an QTRAP 6500 mass spectrometer (SCIEX, Darmstadt, Germany). For data acquisition Analyst version 1.6.2 and for data processing Multiquant version 3.0.3 (both SCIEX) was used. Mass spectrometric and chromatographic operating conditions were chosen according to Gerding et al. ([Gerding et al. 2015](#_ENREF_3)) and Osteresch et al. ([Osteresch et al. 2017](#_ENREF_4)), respectively.
