# Supplementary material for: Mycotoxins in blood and urine of Swedish adolescents—possible associations to food intake and other background characteristics
Source: Mycotoxin Res. 2019 Dec 14;36(2):193–206. doi: 10.1007/s12550-019-00381-9 (PMC7182616; doi:10.1007/s12550-019-00381-9)
Supplement: Supplementary file 3 — (DOCX 17 kb) [file 12550_2019_381_MOESM3_ESM.docx]

**Electronic Supplemental Material 4**. Mean concentration of total DON equivalents (DON + DON-15GlcA) in all urine samples adjusted for density or creatinine

| Grade | Sex | Number | DON eqv. density adjusted (ng/ml)±s.d. | | | Number^1^ | DON eqv. creatinine adjusted (ng/ml)±s.d. | | |
| --- | --- | --- | --- | --- | --- | --- | --- | --- | --- |
|  |  |  | LB^2^ | MB^3^ | UB^4^ |  | LB | MB | UB |
| 5 | Female | 165 | 1.50±4.91 | 2.78±4.85 | 4.06±4.91 | 157 | 1.86±7.31 | 3.02±7.17 | 4.19±7.04 |
|  | Male | 166 | 2.63±13.8 | 3.81±13.6 | 5.00±13.5 | 160 | 1.14±5.37 | 2.31±5.31 | 3.49±5.27 |
| 8 | Female | 232 | 1.65±9.30 | 3.07±9.16 | 4.48±9.12 | 224 | 1.26±5.75 | 2.43±5.68 | 3.60±5.63 |
|  | Male | 179 | 1.66±8.67 | 2.95±8.58 | 4.25±8.57 | 170 | 1.16±5.09 | 2.37±5.06 | 3.58±5.07 |
| 11 | Female | 219 | 1.81±8.92 | 3.45±8.80 | 5.10±8.87 | 210 | 1.72±12.3 | 2.90±12.2 | 4.08±12.1 |
|  | Male | 135 | 1.30±4.87 | 2.76±4.85 | 4.21±5.05 | 123 | 3.65±12.7 | 4.80±12.5 | 5.95±12.2 |
| **Total** | all | 1096 | 1.77±9.03 | 3.16±8.93 | 4.55±8.92 | 1044 | 1.69±8.58 | 2.87±8.47 | 4.04±8.37 |

^1^Samples with creatinine levels < 0.3 or > 3.0 mg/ml are deleted according to Cocker et al. ([Cocker et al. 2011](#_ENREF_1))

^2^LB: Lower bound: Values below LOD and LOQ are replaced with 0

^3^MB: Middle bound: Values below LOD and LOQ are replaced with half of their respective values

^4^UB: Upper bound: Values below LOD and LOQ are replaced with their respective values
